# Multiscale modeling methods in biomechanics

**DOI:** 10.1002/wsbm.1375

**Published:** 2017-01-19

**Authors:** Pinaki Bhattacharya, Marco Viceconti

**Affiliations:** ^1^ Department of Mechanical Engineering and INSIGNEO Institute for *in silico* MedicineUniversity of SheffieldSheffieldUK

## Abstract

More and more frequently, computational biomechanics deals with problems where the portion of physical reality to be modeled spans over such a large range of spatial and temporal dimensions, that it is impossible to represent it as a single space–time continuum. We are forced to consider multiple space–time continua, each representing the phenomenon of interest at a characteristic space–time scale. Multiscale models describe a complex process across multiple scales, and account for how quantities transform as we move from one scale to another. This review offers a set of definitions for this emerging field, and provides a brief summary of the most recent developments on multiscale modeling in biomechanics. Of all possible perspectives, we chose that of the modeling intent, which vastly affect the nature and the structure of each research activity. To the purpose we organized all papers reviewed in three categories: ‘causal confirmation,’ where multiscale models are used as materializations of the causation theories; ‘predictive accuracy,’ where multiscale modeling is aimed to improve the predictive accuracy; and ‘determination of effect,’ where multiscale modeling is used to model how a change at one scale manifests in an effect at another radically different space–time scale. Consistent with how the volume of computational biomechanics research is distributed across application targets, we extensively reviewed papers targeting the musculoskeletal and the cardiovascular systems, and covered only a few exemplary papers targeting other organ systems. The review shows a research subdomain still in its infancy, where causal confirmation papers remain the most common. *WIREs Syst Biol Med* 2017, 9:e1375. doi: 10.1002/wsbm.1375

For further resources related to this article, please visit the WIREs website.

## INTRODUCTION

**Figure 1:**
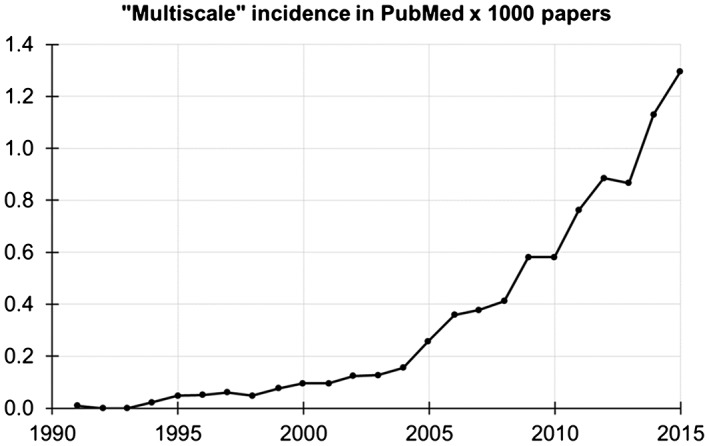
Incidence of multiscale papers indexed in PubMed from 1991 to 2015. Incidence is obtained by dividing for each year the number of papers retrieved with the search ‘Multiscale [ALL]’ by the total number of papers indexed in that year.

As per March 2016, PubMed indexed 2180 papers including the word ‘multiscale’ in the title, and 5457 anywhere in the PubMed record. While the first of these papers was published in 1979, it is only in the last ten years that the biomedical research community has started to think across scales (Figure 1). Biomechanics research follows similar trends.

The aim of this study is to provide a systematic review of the multiscale modeling methods reported so far in biomechanics research. It also aims to offer a set of candidate definitions for this emerging field. As a lot of multiscale biomechanics involves either the musculoskeletal or the cardiovascular system, we will systematically review these two specific areas. However, we will also provide an overview of other interesting applications.

### Definitions

The definition of *scale* varies widely depending on the context; in its simplest instance, it can be defined in term of grain and extent, both in space and time. The grain can be defined as largest value between the lower limit of spatial/temporal resolution allowed by the instrumentation, and the smallest/fastest feature of interest to be observed. Similarly, the extent can be defined as the smallest value between the upper limit of spatial/temporal resolution (i.e., the volume of interest in a four‐dimensional space) and the size of the largest/slowest feature of interest to be observed. Resolution is defined as ‘the smallest interval of a measured signal that will still cause a change in the measurement result.’^1^ In a perfect world, we would not need to worry about scales, because we would be free from the ‘curse of resolution.’^1^ Because our ability to resolve quantities in space and time is limited, ‘to explore from the infinitely small to the infinitely large with a finite resolution we need scales.’^1^

Most engineering theories avoid this complexity through one fundamental, and often implicit, assumption: *scale separation*. In a steel beam the microstructure grain size is 10^5^ times smaller than the beam length. This means that every small portion of the macrostructure contains thousands of microstructural elements, whose properties can be described statistically within that small volume. In other words, we do not need to have a detailed description of the microstructure to calculate the mechanical behavior of the macrostructure, but only its average properties as they manifest at the macroscopic scale. In every biological material of interest in biomechanics research, the degree of scale separation is much smaller, typically around 10^2^, so making this assumption is much less safe.

From a computational point of view, it is frequently more convenient to build a multiscale model not as a monolithic object, but rather as the orchestration of multiple models, each typically capturing the causal relation at a given space–time scale. In this case we refer to each single‐scale model as a *hypomodel*, and to their orchestration as a *hypermodel*.^2^

Also in some implementations it is more convenient to separate the models that capture the causal relation at each space–time scale, from those that capture the transformation on the quantities involved from one scale to another (sometimes referred to as *homogenization*, when they transform from small to large, and *particularization*, when they transform from large to small). In this case, we call *component* models those capturing single‐scale causation, and *relation* models those capturing the scale transformation.

For the purpose of this study, we define a model as any causal quantitative relation M between an input set I and an output set O, so that:

$$O^{\prime }\;=\;M\left(I\right)$$

In the physical and natural sciences M captures some knowledge about nature; such knowledge can be phenomenological (purely based on induction, i.e., exclusively on experimental observations), or mechanistic (based on deduction, i.e., on theoretical reasoning), although in practice ‘both phenomenological and mechanistic approaches are inherently present in any model.’^3^ The variable I represents a set of *necessarily* measurable quantities, whereas O′ is the prediction of a set of *desirably* measurable quantities O.

As most of biomechanical models tend to be complex, most often M(I) is not computable in closed form, and we need to resort to some numerics N:

$$O^{\prime \prime }\;=\;N\left(M\left(I\right)\right)$$

O″ differs from the true value O for three reasons: (1) the *approximation* due to N; (2) the *aleatory uncertainty* associated with the measurement of I (and if possible O); and (3) the *epistemic uncertainty* associated with the model M. We use *verification*, *uncertainty quantification* (also called sensitivity analysis), and *validation* (when a measure of O is available) methods respectively to estimate these three sources of predictive inaccuracy.^4^

### Structure

The biomechanical multiscale modeling literature is highly heterogeneous, and presents regularities only if you compare papers dealing with the same problem. This makes any attempt to review the overall field quite challenging. In the following we will review the relevant literature along two dimensions. First, we divide papers by organ system; we review in detail papers targeting the musculoskeletal and the cardiovascular systems, by far the most popular in the literature. However, we offer only an overview, without any pretence of being exhaustive, for the other organ systems.

The second and more important dimension we used in this review is that of the modeling intent, expressed in terms of *operational motivations*. Reviewing the literature, we found that of all differences this is the most profound, as it severely influences the difficulty of the challenge involved. But what are the operational motivations that require the building of models where I and O are defined at radically different space–time scales? We have identified three common motivations:

1. *Causal confirmation*. When we need a causal explanation of why O is observed given I, and O and I are defined on different space–time scales, the experimental testing of causal hypotheses can be quite challenging. In these cases, multiscale models are used as materializations of the causation theories, whose falsification is attempted by measuring a large set of I and O values (possibly independently), and then by comparing the range of observed O values with the range of O″ values predicted by the model for the range of I values. If the range of O and O″ differ considerably, this means that the causal theory the model materializes is not compatible with the observations, which ‘falsifies’ such a theory.
2. *Predictive accuracy*. In all problems where we need to know the quantity O with a given accuracy, O is difficult to measure directly, but other measurable quantities are known to be causally related to O, we can use that causal knowledge to build a model that predicts O given some other measurable quantities I. In many cases, we can obtain accurate predictions using a causal knowledge entirely defined with a single space–time scale. However, in some cases the only way to obtain the necessary accuracy is to stop assuming scale separation, and extend the predictive model to account for causation across multiple scales.
3. *Determination of effect*. In other problems, the need for a multiscale model does not come from the desire of improving the predictive accuracy of O, but rather from the need to develop a predictor of the effect of quantity I defined at the scale S1 on the quantity O defined at the scale S2.

As these motivations are fairly different, and this reflects deeply on the approach used to develop and evaluate multiscale models in the following, we will review the literature by highlighting which of these three aims is pursued in each paper.

The vast majority of the body of multiscale modeling research has been motivated by *causal confirmation*. In the following we briefly review these studies, and emphasize the relatively fewer studies which went beyond *causal confirmation* and were motivated by *predictive accuracy* or *determination of effect* in their modeling efforts.

## MULTISCALE MODELS OF THE MUSCULOSKELETAL SYSTEM

The musculoskeletal system is considered herein to comprise of the following tissue types: bone, cartilage, skeletal muscle (i.e., excluding smooth muscle and cardiac muscles), tendon, and ligament. Without any pretence to being exhaustive, we highlight below recent advances relating to bone mechanics, bone adaptation and remodeling, fracture healing, skeletal muscle remodeling, electromechanical behavior of skeletal muscle, tendon mechanics, tendon remodeling, and the mechanics of tendon under conditions of homeostasis and pathological mineralization.

### Causal Confirmation

#### *Bone Mechanics*

The goal of predicting whole bone mechanics from information on bone material and structural composition is motivated by its clinical relevance in fracture prediction. That a relationship between apparent scale mechanical response and the material and structural properties at the microscale should exist is intuitively clear. However, the challenge is to base the relationship on microscale variables that are either measurable nondestructively, or are ‘universal’ (not specimen‐specific) in nature. The groundwork for this approach was first laid in the 1990s by Crolet and coworkers^5^, ^6^ following the work on microscale structural and material characterization done in the decades prior (reviewed by Currey^7^). Crolet and coworkers^5^, ^6^ applied the theory of micromechanics^8^, ^9^, ^10^ previously developed for the analysis of engineered composite materials comprising distinct phases (e.g., mineral, matrix, and voids). Causal confirmation of multiscale models based on such homogenization approaches was demonstrated in the works of Hamed *et al*.,^11^ Martinez‐Reina *et al*.,^12^ and Sansalone *et al*.^13^

#### *Bone Adaptation and Remodeling*

The process of natural bone adaptation (bone remodeling) is driven by underlying cellular processes which are in turn influenced by biochemical and mechanosensitive activation. In modeling bone adaptation, one approach is to explicitly account for bone remodeling although mathematical models of bone remodeling are themselves relatively untested.^14^ Various authors adopted a multiscale modeling approach,^15^, ^16^, ^17^ implementing analytical scale‐bridging relationships from the mineral constituent scale through the bone tissue scale along with a cell‐scale bone remodeling algorithm. Due to challenges in measuring bone remodeling activity parameters in a specimen specific manner, the above model predictions relating to the evolution of bone mineral content with time could only be compared against a micro finite element simulation with identical initial bone mineral content and cellular activity parameters. Hambli^18^ introduced a multiscale model that coupled a finite element model at the whole bone scale to a neural network surrogate model at the tissue scale which was trained using micro FE simulations on high‐resolution image‐based models of cancellous bone. Thus, the mechanobiology approach to bone adaptation has yielded only causal confirmation to date.

A different approach to bone adaptation is based on the hypothesis that local microarchitecture of bone is governed by a material redistribution problem that seeks to simultaneously minimize material used, maximize resistance to applied loading, and control bone surface area and permeability. This approach—which is blind to the details of the cell‐scale bone remodeling process—was first proposed in Fyhrie *et al*.^19^ Coelho *et al*.^20^ recently advanced this approach by implementing a multiscale model where at selected locations of the bone, microscale material redistribution problems were solved given the state of stress transferred from the bone scale due to physiological activity (normal walking and stair climbing). The resulting steady state periodic microstructures were used to compute the apparent scale density and orthotropic elastic stiffness tensor components. The predicted bone density distributions, and the power law relationship between predicted local bone density and predicted local elastic tensor components agreed in general with those reported in literature, suggesting that the causal theory the model represented was at least compatible with the range of available observations. Thus, this line of research has also yielded only causal confirmation.

#### *Fracture Healing*

Multiscale models of osteogenesis in order to predict fracture healing are another line of research where predictive accuracy is yet to be demonstrated. The multiscale model of Carlier *et al*.^21^ combined earlier models of DII4/Notch signaling at the intracellular scale^22^ and a bio‐regulatory framework of angiogenesis.^23^ At the intracellular process time‐scale, the model predicts tip cell movement and sprout formation. At the tissue‐level time scale, the integrated effect of angiogenesis and associated transport of molecules regulates differentiation and proliferation of various cell types (e.g., mesenchymal stem cells, chondrocytes, osteoblasts, and fibroblasts) toward bone formation. The multiscale model is exercised with input obtained from literature sources and its predictions qualitatively match experimental observations of temporal evolution of bone, cartilage and fibrous tissue types in a rodent model. This model was enhanced with a more detailed oxygen budget model,^24^ in order to improve the qualitative agreement of the model predictions of cartilage tissue temporal evolution for a large defect in a rodent model with the available experimental observations. In Carlier *et al*.,^25^ the model predictions showed qualitative agreement with regard to spatial distribution of tissue types and steady‐state union/nonunion outcomes.

#### *Skeletal Muscle Remodeling*

Similar to the mechanobiology approach to bone remodeling, the remodeling and adaptation of skeletal muscle has received considerable attention (see the recent review by Wisdom *et al*.^26^). Although the microscale architecture of the muscle and the process of force generation in the muscle (active/passive) are reasonably well understood, the mechanosensitivity of the muscular remodeling process is largely phenomenologically defined.^27^, ^28^ Zöllner *et al*.^29^ obtained causal confirmation for a multiscale model that predicted the shortening of the gastrocnemius muscle as a result of remodeling induced by high‐heeled footwear use. In their model, the apparent scale muscle length was a function of cellular scale sarcomere number. The evolution of sarcomere number with time was dependent on physical activity level represented by a strain‐threshold (cf. physical activity level is represented by a strain energy density threshold in bone remodeling).

#### *Tendon Homeostasis*

Biochemical and biomechanical factors that affect Achilles tendon homeostasis were reviewed by Smith *et al*.,^30^ who pointed out the significant gaps in knowledge regarding tendon structure and function. The paper proposes a conceptual framework that encapsulates collagen fibril–fiber hierarchical organization; the relevance of crimps to tendon damage; and various regimes of tendon repair that included or excluded inflammatory response. Maceri *et al*.^31^ implemented a multiscale model for the tendon to predict: (1) its mechanical response; (2) its remodeling in response to physical activity; and (3) strains within a coupled muscle model in response to coupled neuromuscular excitation. At the tendon‐scale, lumped‐parameter models were used to describe viscous response and strain‐dependent elastic response in the tendon. The parameters at the tendon scale were determined from homogenization of properties at the tissue‐scale (e.g., fiber aspect ratio, fiber curvature, and fiber tangent modulus). Tissue‐scale properties were derived from persistence length, contour length, kink dimension, and end‐to‐end reference length at the molecular scale. The study explores causal confirmation for the proposed multiscale model by comparing predictions with range of values reported in literature.

#### *Mineralized Tendon Mechanics*

Avian tendon tissue is known to mineralize under physiological conditions. Yet, as a partially mineralized soft tissue mineralized turkey leg tendon (MTLT) serves as a model to understand pathological mineralization of human tendon tissue. Spiesz *et al*.^32^ modeled the indentation modulus of a microporous collagen fibril array at the tissue‐scale by employing a Mori–Tanaka homogenization of the nanoscale variables fibril volume fraction, the mineral distribution between fibrils and extra‐fibrillar matrix and the degree of mineralization. Fibril volume fraction and mineral volume fraction were measured in the same study from two distinct tissue zones (circumferential and interstitial) each from a tendon sample. The parameter controlling mineral distribution between fibrils was varied within the range of values in literature. Other model parameter values were taken from literature. Distinct mineral distribution parameter values for circumferential and interstitial tissue regions were found to fit satisfactorily the measured indentation moduli. In a later study,^33^ the same group found that using a single average value of the mineral distribution parameter resulted in the variation of microindentation moduli explained by the tissue‐scale model to be higher in the circumferential zone of the tendon (*R* ^2^ = 0.231) than in the interstitial zone (*R* ^2^ = 0.003). An independent measurement of the mineral distribution parameter is needed to better validate the model.

**Figure 2:**
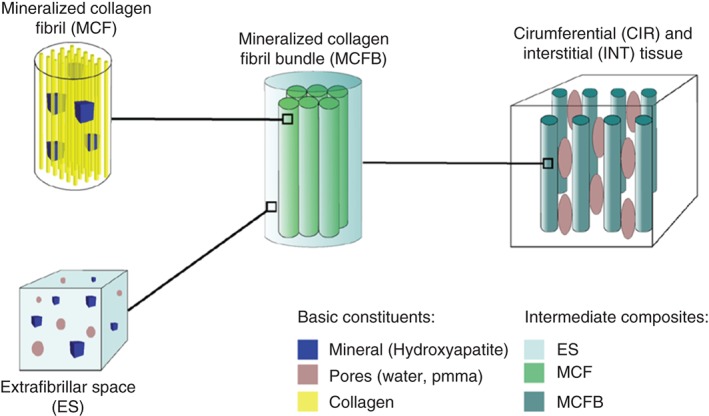
Model hierarchy of MTLT (CIR and INT) tissues. (Reprinted with permission from Ref 34. Copyright 2014 Springer‐Verlag)

Tiburtius *et al*.^34^ identified separate circumferential and interstitial tissue zones (Figure 2) and employed homogenization methods (to bridge across length‐scales) and hierarchical organization along the lines proposed by Spiesz and coworkers.^32^, ^33^ Tiburtius *et al*.^34^ experimentally determined microporosity, degree of mineralization, and acoustic impedance for a number of tendon samples. Model parameter values not directly measured were varied in the range reported in literature to determine their influence on the sensitivity of the tissue‐scale stiffness tensor for each sample. The stiffness tensor was used to derive an effective acoustic impedance. The study found that the multiscale model captured the separation of acoustic impedances between circumferential and interstitial tissue zones over the range of measured mineral volume fraction in fibril bundles. Furthermore, the variation of computed acoustic impedance matched the variation of measured acoustic impedance—in both order of magnitude and trend—over the range of measured mineral volume fraction in fibril bundles, thus confirming the causal basis in their model.

### Open Problems Motivated by Predictive Accuracy

For each multiscale model in musculoskeletal (MSK) biomechanics that is motivated by causal confirmation there exists an open problem that is motivated by predictive accuracy (PA), which has a stricter demand in terms of model validation. We offer one open problem each for the six applications discussed in this section:

**MSK_PA1**. Bone mechanics: A multiscale model to predict the elastic anisotropy as measured in a given bone tissue specimen, using structural and composition information taken at lower scales from the same specimen
**MSK_PA2**. Bone remodeling: A multiscale model to predict the evolution of bone mineral content as measured in a given bone volume, using bone remodeling activity parameters measured on the same specimen
**MSK_PA3**. Fracture healing: A multiscale model to predict tip cell movement and sprout formation as measured within a bone fracture site volume, using intracellular and tissue scale parameters measured on the same specimen
**MSK_PA4**. Skeletal muscle remodeling: A multiscale model to predict the shortening of the gastrocnemius muscle as measured on a subject, using sarcomere scale parameters measured on the same subject
**MSK_PA5**. Tendon homeostasis: A multiscale model to predict whole tendon remodeling as measured on a subject, using tissue and molecular scale measured on the same subject
**MSK_PA6**. Mineralized tendon mechanics: A multiscale model to predict the tissue stiffness tensor measured on a tendon specimen, using structural and composition information obtained on the same specimen

### Predictive Accuracy

#### *Bone Mechanics*

**Figure 3:**
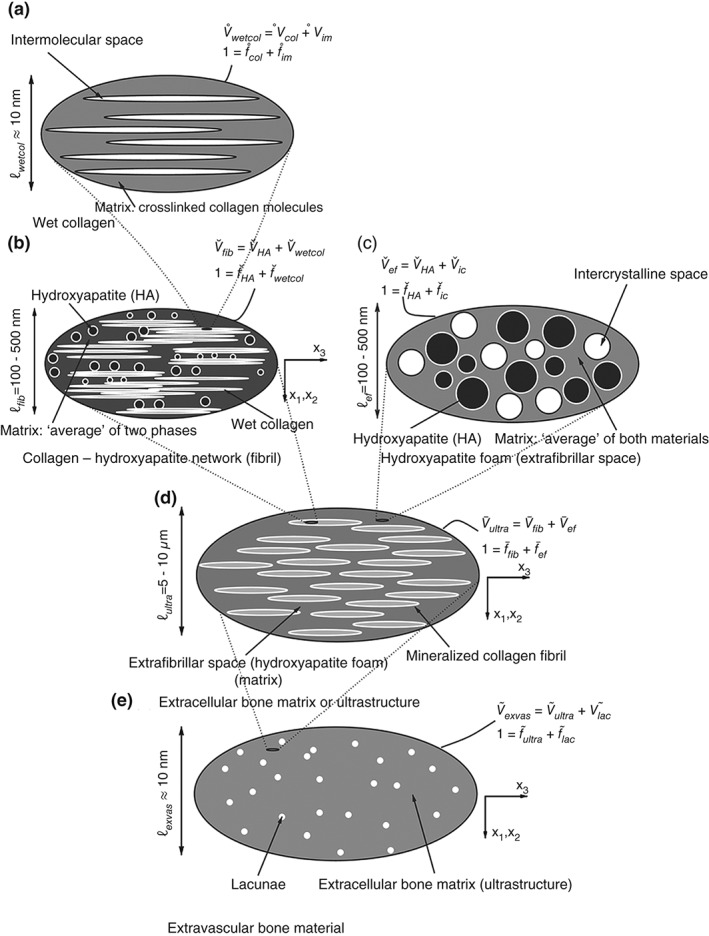
Micromechanical representation of bone material by means of a five‐step homogenization procedure. (Reprinted with permission from Ref 36. Copyright 2007 Elsevier)

Predictive accuracy of multiscale modeling of bone mechanics was demonstrated in the work of Hellmich and coworkers^35^, where the bone material is considered a hierarchically organized composite of hydroxyapatite (HA) crystals, collagen and water (Figure 3). The elastic properties of the basic constituents were shown to be ‘universal’ and were determined from separate experiments. Hellmich *et al*.^35^ showed that this multiscale model can predict bone stiffness given only the information on volume fraction of each constituent. The study assessed the predictive accuracy of the multiscale model by comparing individually the experimentally measured stiffness for multiple cortical and trabecular bone specimens with the stiffness predicted using their multiscale model.

Fritsch *et al*.^36^ showed that the multiscale model could use the identical specimen‐specific information of volume fraction of the constituents and accurately predict mass density of bone regions smaller than the tissue scale, for example, extracellular and extravascular bone regions. At the same time, considering the inclusions in the bone composite to possess a given distribution of orientations, Fritsch *et al*. successfully assessed the predictive accuracy of the model with respect to tissue scale elastic anisotropy, thus directly answering the problem MSK_PA1 posed earlier. The same group, in a follow‐up paper^37^ added to the above multiscale model a description of postelastic response at the micrometer scale: brittle rupture of collagen cross‐links and an ideal plastic yielding of the mineral crystals. Satisfactory predictive accuracy of tissue‐scale strength was reported considering cortical bone regions from human and bovine long bones. Eberhardsteiner *et al*.^38^ included in the above model nanoscale sliding of mineral crystals over water layers in order to explain observed viscoelasticity of wet and dry bone tissue specimens and assessed the model accuracy against specimen‐specific experimental observations.

#### *Skeletal Muscle Electromechanics*

**Figure 4:**
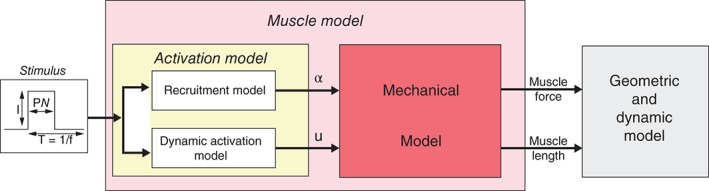
Complete model of the muscle exhibits three blocks. (Reprinted with permission from Ref 41. Copyright 2011 Springer‐Verlag)

Models of the electromechanical behavior of skeletal muscle can be used to assess the risk of muscular degeneration,^39^ or to better design functional electrical stimulation interventions used in treatment and rehabilitation.^40^ El Makssoud *et al*.^41^ introduced a multiscale model of the electromechanical behavior of skeletal muscle under isometric conditions (i.e., in the absence of limb movement) (Figure 4). The multiscale model comprises the whole muscle, the muscle fiber, and the sarcomere length scales. Motor units (MUs) attached to muscle fibers are either contracting, relaxing, or in a completely relaxed state. The relative fraction of MUs in each state is determined by a recruitment model that accounts for the applied electrical stimulation. The relative contraction of given muscle fiber is assumed to result in an identical relative contraction in each component sarcomere.

The sarcomere model^42^ determines the stiffness and force generated by a sarcomere as a consequence of the applied electrical stimulation and relative contraction. The model homogenizes across the whole‐muscle/muscle‐fiber length scales by assuming identical mechanics for all muscle fibers in a given state (contracting, relaxing, or completely relaxed). The predictive accuracy of the recruitment model was tested against published experimental measurements.^43^ It was also possible to find a single set of parameters for the multiscale model that could predict the whole muscle scale force in a rabbit knee experiment under two different electrical stimulation conditions. Finally, a different set of model parameters could also be found corresponding to the prediction of whole muscle scale forces in patients with spinal‐cord injury.^44^

#### *Tendon Tissue Mechanics*

As a resolution to the problem MSK_PA6 posed earlier, Szczesny and Elliot, in two consecutive papers,^45^, ^46^ reported predictive accuracy for their multiscale model of a tendon fascicle comprising discontinuous, staggered, crimped fibrils. A probability distribution informed the number of fibrils that were fully un‐crimped at a given level of stretch in the tendon; the load supported by each fibril was taken to be nonzero only in the un‐crimped state; the fibril load was transferred to an interfibrillar interface possessing a perfectly plastic response initiated at incipient sliding. The model was analyzed using microstructural parameter values (radius, length and volume fraction of fibrils) from the literature and micromaterial parameters (interfibrillar interface plastic stress, fibril modulus and fibril stretch distribution) from experiments conducted by Szczesny *et al*.^46^ The model predicted accurately the ratio of strains in the fibril and the tendon over a range of tendon‐strains in rat‐tail tendon fascicles.^46^ Over this range of tendon strain, the tendon undergoes plastic unloading and the plastic response was satisfactorily captured due to the fibril‐scale plasticity included the multiscale model. The results suggested that it was the plasticity of the interfibrillar interface rather than that of the fibril itself, which resulted in the accurate prediction.^45^ The study considered an elastic regime of the interfibrillar interface up to a finite sliding distance, but showed that the resulting elastoplastic micromechanics improved the prediction only up to relatively small tendon strain values.^45^

### Open Problems Motivated by Determination of Effect

As before, we pose open problems in multiscale modeling in musculoskeletal biomechanics motivated by *determination of effect* (DE).

1 **MSK_DE1**. Bone mechanics: A multiscale model that predicts better than a single‐scale model the bone tissue elastic anisotropy
2 **MSK_DE2**. Skeletal muscle electromechanics: A multiscale model that predicts better than a single‐scale model the force–length relationship in a muscle
3 **MSK_DE3**. Tendon tissue mechanics: A multiscale model that predicts better than a single‐scale model the stiffness tensor of a tendon tissue

### Summary

The discussion in this section showed that most of the work in multiscale modeling of musculoskeletal biomechanics has been motivated by *causal confirmation*. Of the three categories defined in this article, *causal confirmation* imposes the least demand on model validation. Thus, by simply raising the bar on validation to the next level, we posed six open problems motivated by *predictive accuracy*. Two of the six problems were found to have been answered. Table 1 summarizes the multiscale musculoskeletal biomechanics models, that were motivated by *predictive accuracy*. Reflecting on the four problems that remain open (MSK_PA 2–5), it is evident that multiscale modeling of musculoskeletal biomechanics problems involving cellular remodeling/adaptation processes remains a challenge. Finally, we offered three additional open problems motivated by *determination of effect*.

**Table 1:** Summary of Multiscale Musculoskeletal Biomechanics Models

Application	*Scale S1*	*Scale S2*	*Other Interacting Scales*	Component Hypomodels	Relation Hypomodels
	I	I	I		
		O			
Bone tissue mechanics	*Wet collagen*	*Extravascular bone (tissue)*	*Fibril; extracellular bone matrix*	Elasticity, yield, viscoelastic creep models at each scale	Homogenization models (e.g., Mori–Tanaka)
	Volume fractions, elasticity, viscosity of water and collagen, shape of intermolecular spaces	Volume fraction and shape of lacunar spaces, elasticity of extracellular bone matrix	Volume fractions, inclusion shapes, elasticity and creep of wet collagen, HA crystals, intercrystal‐line void spaces and collagen fibrils		
		Stiffness, anisotropy, strength, viscoelasticity			
Skeletal muscle electromechanics	*Sarcomere*	*Whole muscle*	*Muscle fiber*	Models for sarcomere mechanics, fiber activation and mechanics, muscle recruitment and mechanics	Models for cross‐bridge distribution in sarcomere controlled by muscle activation, for affine stretch transformation between fiber and sarcomere scales, for averaging of fiber response to obtain muscle response
	Cross‐bridge attachment and detachment rates, rest length and stiffness	Fiber recruitment states, lumped mechanical model parameters: rest length, stiffness, mass, damping	Electrical stimulation, activation model parameters		
		Muscle force			
Tendon mechanics	*Fibril; interfibrillar matrix*	*Fascicle (tendon tissue)*		Fibril and interfibrillar matrix elasto‐plasticity laws	Shear lag model to homogenize between fibril and fascile
	Radius, length, elastic modulus of fibril; interfibrillar matrix stiffness and yield parameters	Volume fraction of fibrils, probability distribution of fully uncrimped fibrils			
		Fascicle elasto‐plasticity			

For each application (first column), the following scales are detailed in columns 2–4: the smallest scale S1, the scale S2 at which model prediction is desired, all other scales. For each scale, the inputs (I) used in the multiscale model are listed. For scale S2, the set of variables to be predicted (O) are also listed. For each multiscale modeling application, the component models at each scale, and relation models between the scales, are indicated in the last two columns.

## MULTISCALE MODELS OF THE CARDIOVASCULAR SYSTEM

In this section, we consider research on the cardiovascular system, for example, studies on heart rate and blood flow. Yet, as the vascular system penetrates other organs and organ systems (e.g., brain and lungs), we also include in the discussion studies that seek to model the interaction between vasculature and other organs or organ systems. An exception to this rule is the interaction between vasculature and the musculoskeletal system, which has already been discussed in the foregoing section.

### Causal Confirmation

#### *Blood Flow Interaction with Blood Vessel Walls*

The understanding of the interaction between blood flow and the blood vessel walls is essential for several medical problems such as aneurysms and atherosclerosis.^47^ Although the dysregulation of blood flow or arterial endothelial function is typically restricted to a local region, it influences and is influenced by the flow in regions far away from the site of the pathology. Twenty years ago, Dubini *et al*.^48^ proposed a geometrically multiscale approach to model the multiphysics problem of fluid structure interaction, to which others have added to subsequently.^49^, ^50^, ^51^ In this approach, the local site of interest is modeled in three dimensions using patient‐specific geometry—an area where much progress has been made in the past decade.^52^ For the flow–structure interaction in the three‐dimensional (3D) model both monolithic^50^ and segregated^53^ coupling algorithms have been developed. The 3D model is coupled to a 0D (electrical circuit analogy) or a 1D (network of segments) model of the circulation system, supplemented by proper conditions specified at the interface of the different models.^54^, ^55^, ^56^ The ongoing research focus in this area is on method development motivated by achieving causal confirmation, and much work remains with regard to model validation.^47^, ^52^

#### *Blood Flow Interaction with Blood Coagulation*

Blood coagulates in response to a rupture of a blood vessel that can potentially lead to loss of blood. Coagulation is effected by platelets, which adhere to the site of breakage, by sensing biochemical signals released by the endothelial cells lining the blood vessel. As such, coagulation is a classic bio‐chemo‐mechanical interaction process. It is also a multiscale problem, with individual platelets on the order of microns, while a wound region is upward of the order of millimeters. Recent reviews^57^, ^58^ on the state‐of‐the‐art of multiscale modeling highlight the challenges in the different approaches taken until now. Specifically, Diamond *et al*.^57^ note that top–down approaches such as neural network models miss patient‐specific features while bottom–up approaches such as systems of ordinary differential equations suffer from incomplete knowledge. Sophisticated multiscale models have been developed that integrate submodels for blood flow through the blood vessel and the growing clot, platelet interactions with blood flow and the vessel wall, and for the coagulation pathway.^59^, ^60^, ^61^, ^62^, ^63^, ^64^ Until now, only causal confirmation has been achieved by these models.

#### *Cellular Mechanics in Blood Flow*

Erythrocytes, or red blood cells (RBCs), transport oxygen and other essential nutrients to the various tissues that the vasculature penetrates through in the human body.^65^ This transport functionality depends on the ability of an RBC to undergo large deformations as it passes through the human circulatory system, an ability that is compromised in diseases such as sickle‐cell anemia or malaria.^65^ Hence there has been a steadily growing interest in the modeling of RBC mechanics in response to surface tractions applied on their boundary. The state‐of‐the‐art of computational approaches on this topic was recently reviewed by Li *et al*.^66^

An extreme case of blood flow through a very small opening occurs in the venous sinuses of the spleen. Salehyar *et al*.^67^ employed the above multiscale model to investigate RBC dynamics and internal stresses in the cell during this passage. They postulated that the high deformation mechanics required to pass through the slit‐like sinus can be accurately captured by including the molecule‐scale unfolding dynamics of the spectrin network which were therein modeled as worm‐like chains and ensuring the intactness of intraprotein, interprotein, and protein‐to‐lipid linkages within the RBC. The model captured ‘infolding’ of the RBC membrane in dependence of the initial relative orientation of the RBC and the slit. Comparison with experimental evidence of RBC shape dynamics was not performed in the study.

#### *Vasculogenesis and Angiogenesis*

The transport of biochemical agents through vasculature controls the growth and maintenance of tissue within which the vasculature penetrates. Examples of multiscale studies modeling such control within the musculoskeletal system, for example, osteogenesis and fracture healing and tendon tissue homeostasis and repair, were visited in the previous section. Along similar lines, multiscale models for vasculogenesis and angiogenesis have also been developed. Using a cellular Potts modeling framework, Scianna *et al*. investigated angiogenesis^68^ by detailing the interactions between cellular and molecular scale models, and investigated vasculogenesis^69^ by detailing the interactions between subcellular, cellular and extracellular scale models. The multiscale models parameters in each study were determined from experimental evidence in literature, and model prediction qualitatively agreed with observed characteristics.

Scianna *et al*.^68^ incorporated the influence of vascular endothelial growth factor (VEGF) on cellular signaling by defining a model of VEGF diffusion at the extracellular scale and models simulating subcellular processes in dependence of VEGF concentration. Stefanini *et al*.^70^, ^71^ focussed on the distribution of VEGF receptors, and distinguished specifically the distribution of two VEGF isoforms and their receptors in their multiscale model. Their multiscale models included compartment models for blood and tissue (normal, tumor) and intercompartmental interactions. Stefanini *et al*.^70^ showed that under pathological conditions the distinction between VEGF isoforms and between the local distributions of their receptors influences the eventual signaling cascade. Stefanini *et al*.^71^ obtained qualitative causal confirmation for their model by predicting the clinically observed increase in plasma VEGF following the administration of a VEGF antibody. Bonilla *et al*.^72^ and Terragni *et al*.^73^ implemented deterministic and stochastic models of tumor‐induced angiogenesis.

#### *Cerebral Autoregulation in Cardiopulmonary Bypass*

**Figure 5:**
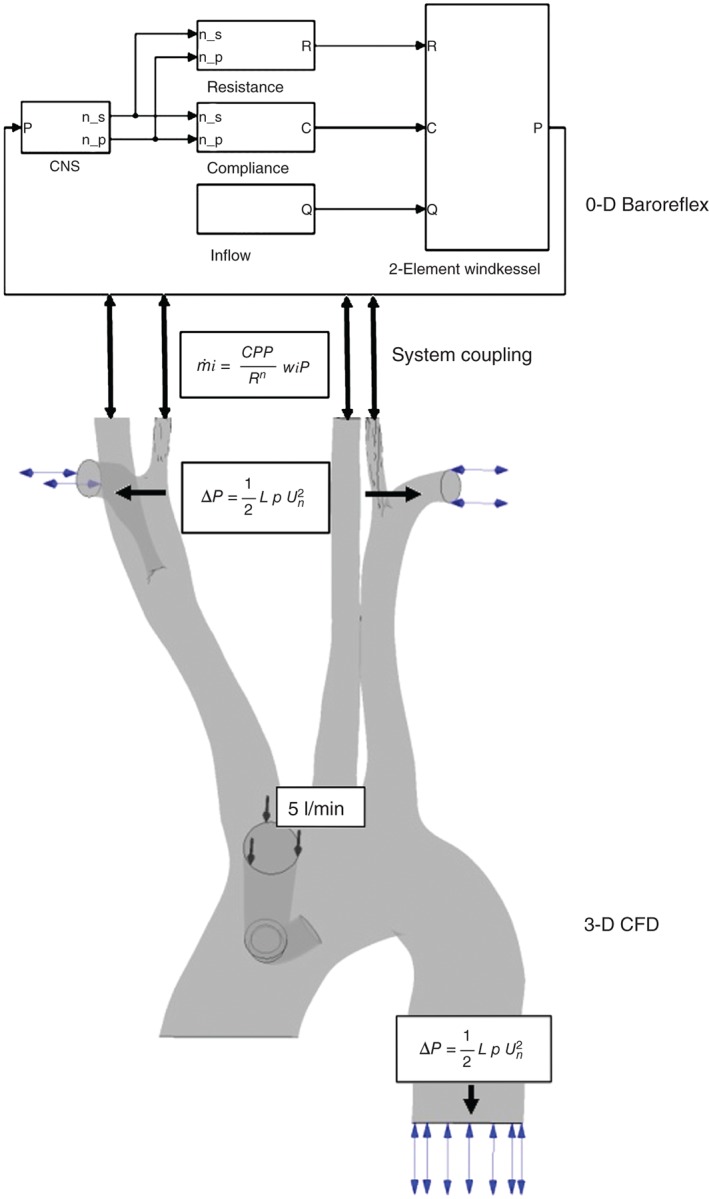
Multiscale model of cardiopulmonary bypass. (Reprinted with permission from Ref 76. Copyright 2014 Elsevier)

The cardiopulmonary bypass (CPB) pump or the ‘heart–lung machine’ is a device routinely used in surgery to take over the function of the heart and the lungs. Neurological malfunction leading to stroke is a common complication of the CPB technique and its prediction has long attracted research interest.^74^ Kaufmann *et al*.^75^ highlighted that the brain's ability to adapt to changing flow conditions during CPB are influenced by the state of its autoregulation mechanism. They also incorporated a phenomenological model for autoregulation within a 3D computational fluid dynamics model for CPB and validated experimentally the cerebral blood flow predictions. Neidlin *et al*.^76^ improved this framework by replacing the phenomenological model with a 0D model of the baroreflex mechanism (Figure 5). The baroreflex model predicted subject‐specific static and dynamic cerebral autoregulation behavior.^76^ This 0D–3D coupling model^76^ was further enriched by Neidlin *et al*.^77^ who included the elasticity of vessel walls in the 3D CPB model this allowing flow–structure interaction. Model predictions of central aortic pressure and blood flow velocity through the descending aorta were compared with experimental observations reported in literature. Causal confirmation for the model was established by the good correspondence of time‐dependent haemodynamic features over one cardiac cycle.

#### *Cardiomyocyte Mechanics*

Weinberg *et al*.^78^ obtained causal confirmation for a model that predicted the influence of high‐frequency stimulation in cardiac myocytes on ionic current amplitude and gating dynamics. Adeniran *et al*.^79^ predicted the difference in the influence of pCa (−log_10_ of the calcium concentration) on force between normal and heart failure with preserved ejection fraction cases as observed by Borbély *et al*.^80^ Gaur *et al*.^81^ provided causal confirmation for a model that predicted stochastic Ca release processes locally in cardiac ventricular myocytes. Hand and coworkers implemented a multiscale model to study the effects of gap‐junctional and ephaptic coupling on conduction,^82^, ^83^ with a microscale description near action potential wave fronts, and a macroscale description in regions far away from them.

#### *Heart Valve Mechanics*

Research interest in the biomechanical function of valves in the different chambers of the heart is motivated by the prediction of diseases such as stenosis and regurgitation and by the need to replace dysfunctional valves.^84^ The mechanics of the heart valves are determined at several scales.^85^ The response of valvular interstitial cells to blood‐flow induced shear stresses can lead to calcification of the tissue and thereby disrupt valve mechanics. Tissue‐scale organization across *fibrosa* and *ventricularis* layers determines the load‐bearing response of the valves at the macroscale. Weinberg *et al*.^85^ reviewed the state‐of‐the‐art experimental and computational investigations into heart valve mechanics at different scales which have led to validated models at each scale. Weinberg *et al*.^86^ introduced a multiscale model where macroscale strains were obtained from flow‐structure interaction of the heart valve. These macroscale strains were applied to a tissue model that was used to determine local tissue‐scale strains in the different layers, and which were used to stimulate a cell‐scale model. Comparing against experimental results reported in literature, causal confirmation was achieved for multiscale predictions of cellular aspect ratio in normal^86^ as well as bicuspid heart valves^87^ and of calcification of the aortic valve during aging.^88^

### Open Problems Motivated by Predictive Accuracy

In a similar manner as before, we pose open problems in multiscale modeling in cardiovascular (CV) biomechanics motivated by *predictive accuracy*.

1 **CV_PA1**. Blood flow interaction with blood vessel walls: A multiscale model to predict the measured blood flow and blood vessel wall dynamics within a local region (e.g., surrounding an aneurysm) of the systemic circulation, using parameters measured on the same circulation system, vascular tissue and geometry, and blood fluid
2 **CV_PA2**. Blood flow interaction with blood coagulation: A multiscale model to predict the measured development of a blood clot, using the bio‐chemo‐mechanical parameters measured on the same platelet–blood–vessel wall system
3 **CV_PA3**. Cellular mechanics in blood flow: A multiscale model to predict the dynamics of an RBC as measured in blood flow, using the micromechanical parameters measured on the same RBC
4 **CV_PA4**. Vasculogenesis and angiogenesis: A multiscale model to predict measured angiogenesis and vasculogenesis based on measured subcellular, cellular and molecular scale parameters
5 **CV_PA5**. Cerebral autoregulation in CPB: A multiscale model to predict the measured cerebral autoregulation, using the measured elasticity of vessel walls on the same cardiac system
6 **CV_PA6**. Cardiomyocyte mechanics: A multiscale model to predict the measured electrical activity in a cardiac myocyte under high‐frequency stimulation, using cell membrane electro‐mechanical property parameters measured in the same myocyte
7 **CV_PA7**. Heart valve mechanics: A multiscale model to predict the change in cell aspect ratio during a cardiac cycle and increase in valvular tissue calcification due to aging, using cellular and tissue‐scale parameters measured in the same human heart valve

### Predictive Accuracy

#### *Cellular Mechanics in Blood Flow*

**Figure 6:**
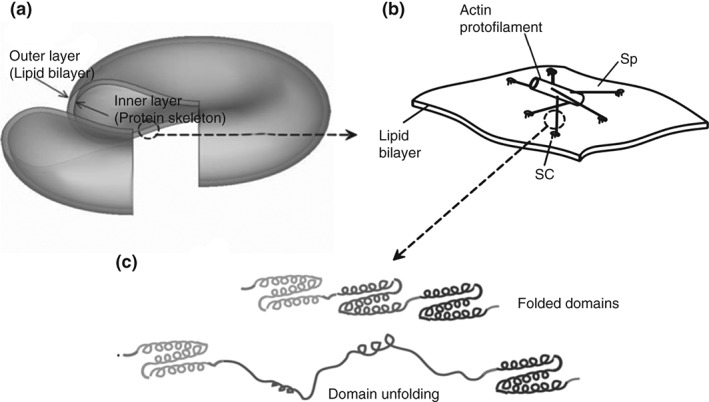
Multiscale model of a red blood cell: (a) complete cell model; (b) molecular‐detailed junctional complex model; and (c) spectrin (Sp) model. (Reprinted with permission from Ref 89. Copyright 2010 American Physical Society)

One approach to the multiscale modeling of RBC mechanics in blood flow, due to Peng *et al*.,^89^ defines three length scales (Figure 6): (1) whole cell scale; (2) junctional‐complex (JC) scale; and (3) spectrin (Sp) protein scale. The protein‐scale submodel^90^ used a worm‐like chain description that was parameterized using measured properties of intraprotein linkages and causally confirmed experimentally observed folding/unfolding dynamics. The JC scale submodel^91^ describes the actin proto‐filament attachments to the RBC lipid bilayer and the Sp network. Finally, in the whole cell scale model^89^ the RBC is defined as a closed shell constituting the protein‐network/lipid bilayer membrane as above and constrained by area and enclosed volume conservation rules. The whole cell model established causal confirmation of the dependence of RBC resting shapes and its microscale properties. Additionally, addressing the problem CV_PA3, this model demonstrated predictive accuracy through comparison with micropipette aspiration and cell stretching experiments.^89^

#### *Autonomic Heart Rate Regulation*

**Figure 7:**
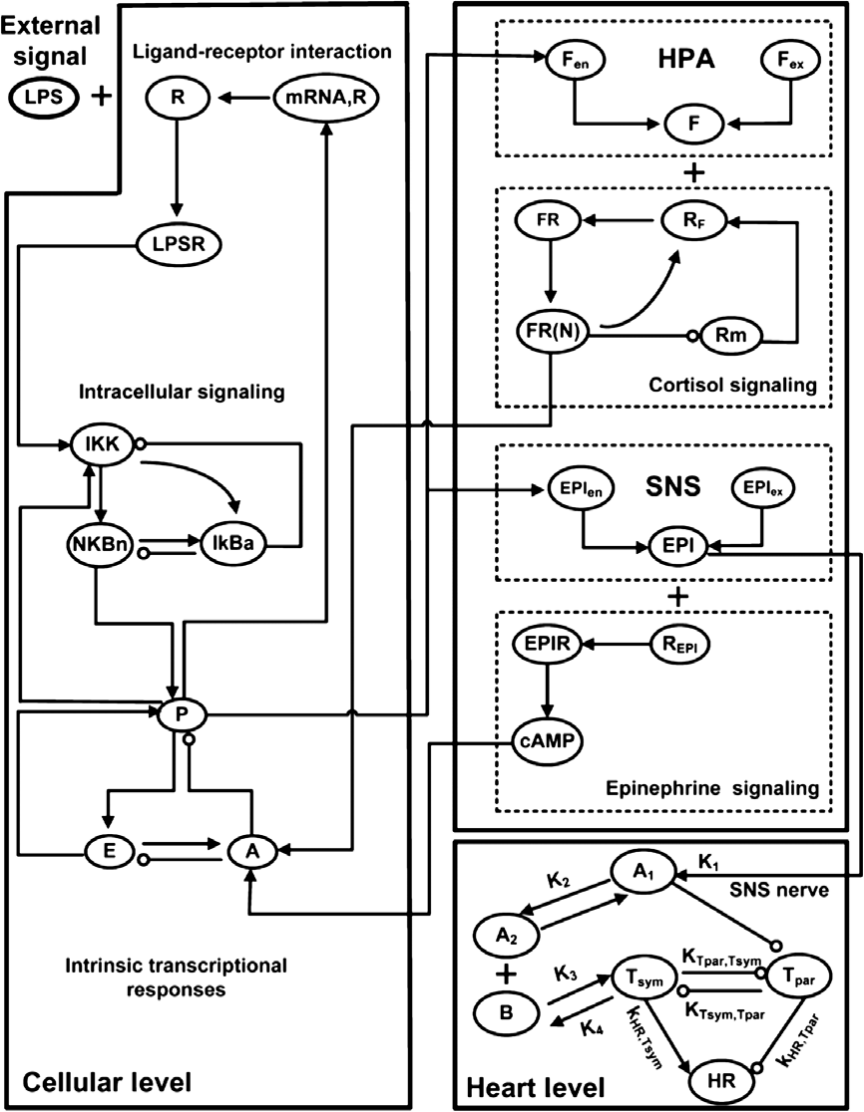
Basic topological interactions composing the multilevel model of endotoxin induced human inflammation. (Reprinted with permission from Ref 95. Copyright 2009 Wolters Kluwer Health, Inc.)

In 1991, the American College of Chest Physicians/Society of Critical Care observed that inflammation response syndrome, or sepsis, was increasingly becoming a ‘cause of morbidity and mortality, particularly in elderly, immunocompromised, and critically ill patients.’^92^ Understanding of the pathogenesis of sepsis has since increased and insights gained about its molecular basis was outlined in a 2005 review by Tetta *et al*.^93^ Foteinou *et al*.^94^ implemented a multiscale model to probe the relationship between systemic inflammation and autonomic heart rate regulation. Their multiscale model comprised three submodels: (1) a cell‐scale model of leukocytes under endotoxin challenge; (2) an organ‐scale model for the heart to determine changes to heart rate variability in response to systemic inflammation; and (3) an organism‐scale neuro‐endocrine system model (Figure 7). Their cell‐scale model is based on a previous description^95^ of endotoxin signaling and associated transcriptional dynamics along with a pharmacokinetics/pharmacodynamics model for exogenous immune‐suppressive agents. At the organ scale, the rate of change of heart rate variability is considered to be a switch‐like function of the cellular pro‐inflammatory response. At the scale of the neuroendocrine system, the model activates the influence of pro‐inflammatory response on cytosol levels at the cell‐scale and defines the influence of the level of epinephrine hormone (secreted by the sympathetic nervous system) on cell‐scale anti‐inflammatory response.

Model parameters associated with the influence of systemic inflammation signaling on cell‐scale transcriptional response are determined from experiments,^94^, ^95^ while parameters associated with the neuro‐endocrine immune system are estimated. The multiscale model predicts accurately the temporal evolution of cortisol concentrations and steroid active signal and the significant differences between conditions of presence and absence of immunomodulatory drug infusion prior to endotoxemia. The model also predicts accurately heart rate variability over time and its relatively low sensitivity to immunomodulatory drug infusion.

Foteinou *et al*.^96^ enriched the above multiscale model by adding the influence of sympathetic and parasympathetic nerve activities on heart rate variability based on a previous model by Warner *et al*.^97^ The additional model parameters are obtained from experiments on human subjects.^96^ This extended model is found to accurately predict experimentally observed temporal changes in parasympathetic activity and heart rate. Furthermore, the model accurately predicts the significant differences in both parasympathetic activity and heart rate between presence and absence of exogenous epinephrine drug infusion prior to the endotoxemic challenge.

#### *Circulation System*

The influence of the venous system on heart dynamics and circulation has attracted the interest of researchers since the late 1960s.^98^ Müller *et al*.^99^ implemented a multiscale model for the circulation system that comprises: (1) a network of major arteries; (2) a network of major veins; (3) lumped‐parameter models for the heart and pulmonary circulation; and (4) lumped parameter models for the arterioles, capillaries, and venules. For the venous submodel, model input parameters are obtained in a subject‐specific manner from experiments. Other submodel predictions are tested against results reported in the literature. Using their multiscale model, Müller *et al*.^99^ demonstrate causal confirmation for predictions of blood flow in the aorta, blood flow in the major leg arteries, blood flow in arteries located in the neck and the head, and blood flow in systemic veins located outside the neck and the head. Furthermore, Müller *et al*.^99^ obtain significant predictive accuracy for subject‐specific blood flow in the veins in the head and in the neck when compared against phase‐contrast MRI data.

### Open Problems Motivated by Determination of Effect

In a similar manner as before, we pose open problems in multiscale modeling in cardiovascular (CV) biomechanics motivated by *determination of effect* (DE).

1 **CV_DE1**. Cellular mechanics in blood flow: A multiscale model that predicts the dynamics of an RBC in blood flow better than a single‐scale model
2 **CV_DE2**. Autonomic heart rate regulation: A multiscale model that predicts autonomic heart rate regulation under endotoxemic challenge better than a single‐scale model
3 **CV_DE3**. Circulation system: A multiscale model that predicts the venous system in the head and in the neck better than a single‐scale model

### Determination of Effect

#### *Cellular Mechanics in Blood Flow*

**Figure 8:**
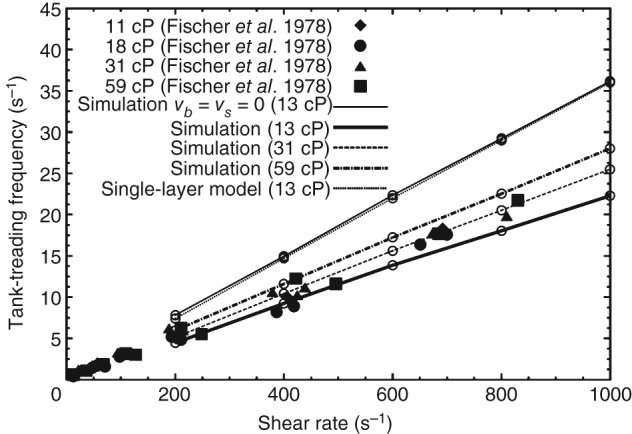
Establishing ‘determination of effect’ of RBC membrane microstructural details on tank‐treading dynamics of RBC in shear flow. ‘Simulation’ refers to the multiscale model^89^, ^90^, ^91^, ^100^ and ‘single‐layer model’ refers to a single‐scale model. The multiscale model simulation with zero membrane viscosity (*v*_*b*_ = *v*_*s*_ = 0) retrieves the single‐scale model result. With a nonzero membrane viscosity, the multiscale model compares better with the experimental results. (Reprinted with permission from Ref 100. Copyright 2011 Cambridge University Press)

In a direct response to the CV_DE1 problem, Peng *et al*.^100^ simulated the aggregated flow of RBCs in surrounding blood plasma using a coupled flow–structure interaction model. They compared their multiscale model predictions against those from a single scale model (possessing a continuum description for the RBC membrane and excluding the detailed bilayer–skeleton architecture). This allowed a ‘determination of effect’ of microstructural properties on RBC mechanics in shear flow, particularly during tumbling and tank‐treading behavior (Figure 8). The tumbling rate of the multiscale model^100^ was slightly higher than that of the single scale model, the shear ratios and protein density ratios were locally different along the membrane surface. Tank‐treading frequencies were better predicted by the multiscale model compared to the single‐scale model as the former allowed for nonzero membrane viscosity.

### Summary

The discussion in this section showed that most of the work in multiscale modeling of cardiovascular biomechanics has been motivated by *causal confirmation*. Based on the reviewed literature, seven open problems motivated by *predictive accuracy* were posed. One of these (CV_PA3) was already found answered in the application area of RBC dynamics in blood flow. This application area was found to have successfully modeled a related problem (CV_DE1) motivated by *determination of effect*. This leaves open two multiscale modeling problems motivated by *determination of effect*, related to autonomic heart rate regulation and venous circulation in the head and the neck. Table 2 summarizes the multiscale modeling approaches in cardiovascular biomechanics that successfully solved problems motivated by *predictive accuracy* and *determination of effect*.

**Table 2:** Summary of Multiscale Modeling Approaches in Cardiovascular Biomechanics

Application	*Scale S1*	*Scale S2*	*Other Interacting Scales*	Component Hypomodels	Relation Hypomodels
	I	I	I		
		O			
Heart rate regulation	*Cell (leukocyte)*	*Organ (heart)*	*System (neuro‐endocrine)*	Cellular transcription dynamics model, models for heart rate control, and for HPA and SNS activity	Model for pro‐inflammatory signal from cell to HPA and SNA, for anti‐inflammatory influence of HPA and SNS on cellular processes, and for biochemical input from SNS to the heart
	Endotoxemic signal, parameters controlling pro‐inflammatory pathways and transcriptional response	Biochemical input from sympathetic nervous system (SNS), parameters regulating heart function	Pro‐inflammatory signals, parameters controlling hypothalamic–pituitary–adrenal (HPA) axis and SNS activity		
		Autonomic outflow and heart rate			
Red blood cell mechanics	*Protein molecule (Spectrin)*	*Cell (erythrocyte)*	*Junctional complex (JC)*	Worm‐like chain model for Sp mechanics, spoked hexagon unit cell model for the JC, area and enclosed volume conservation laws for whole cell	Models for mechanical interactions between Sp and JC, for homogenization of JC mechanics to obtain cellular cytoskeleton–bilayer system mechanics
	Stretch, contour length, persistence length of Spectrin (Sp) molecule	Viscoelasticity of the cytoskeleton–bilayer system, enclosed volume and surface area, mechanical interactions with surrounding plasma fluid	Geometry parameters, mechanical properties of the lipid bilayer and the actin proto‐filament		
		Cell resting shapes, response to micropipette aspiration, stretching, tumbling and tank‐treading behavior in shear flow			
Cranial venous circulation	*Organ (heart and pulmonary circulation)*	*System (arterial and venous)*	*Microcirculation (arterioles, venules and capillaries)*	1‐D circulation model, windkessel model for microcirculation	Boundary conditions for flow and pressure at junctions
	Lumped model parameters, flow and pressure boundary conditions, source of circulation forcing in the heart	Network graph, segmental geometry and elasticity, intersegmental boundary conditions on pressure and flow	Lumped model parameters, inlet and outlet flow and pressure conditions		
		Blood flow in head and neck veins			

For each application (first column), the following scales are detailed in columns 2–4: the smallest scale S1, the scale S2 at which model prediction is desired, all other scales. For each scale, the inputs (I) used in the multiscale model are listed. For scale S2, the set of variables to be predicted (O) are also listed. For each multiscale modeling application, the component models at each scale, and relation models between the scales, are indicated in the last two columns.

## MULTISCALE MODELS OF OTHER BIOMECHANICS PROBLEMS

While a lot of multiscale modeling research targets cardiovascular or musculoskeletal biomechanics, there are some other interesting biomechanics applications that are worth mentioning, without any pretence of being exhaustive.

In respiratory biomechanics, multiscale models are being used to provide ‘… with accurate spatial relationships between airway, vessel and the tissue to which they are tethered,’ necessary for the computational analysis of ‘airway–vessel–tissue interactions such as coupling of ventilation distribution in ‘embedded’ airway models to the large deformation of the lung tissue.’^101^ These models are also used to look at the transport mechanisms,^102^ or to investigate bronchoconstriction.^103^

**Figure 9:**
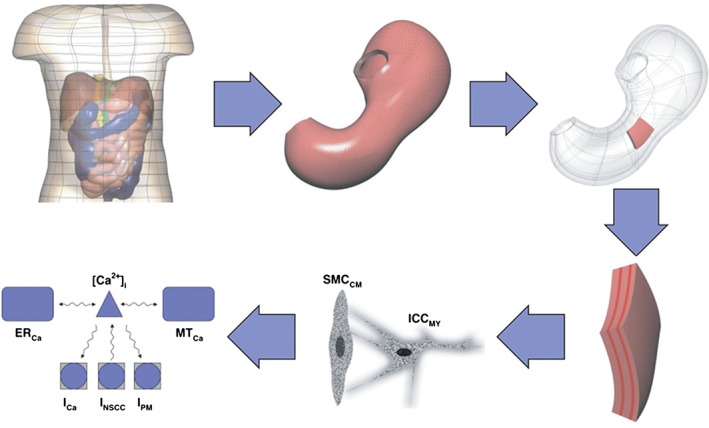
Different spatial scales identified in the modeling of the gastrointestinal system. (Reprinted with permission from Ref 104. Copyright 2010 Wiley)

The gastro‐intestinal system is comprised of organs (stomach, small intestine, and large intestine) that differ significantly in function, organ‐scale morphology and organization at the tissue and cellular scales. In order to achieve proper motility of ingested material through this complex system, muscular activity must be under strong spatiotemporal regulation. This regulation is achieved by gastro‐intestinal electrophysiology.^104^ Du *et al*. provides an extensive review of the multiscale modeling of the gastro‐intestinal tract up to 2010.^105^ The same authors proposed an electromechanical model for the interpretation of electrogastrograms;^106^ more recently, a multiscale model was used to investigate reflux in adenocarcinoma.^107^ In current multiscale models (Figure 9), muscle cell electro‐physiology is modeled either by the phenomenological Aliev *et al*.’s model,^108^ or more recently, the biophysically based model of Corrias *et al*.^109^ These models describe wave equations in transmembrane potential and slow current variables for various ion gating mechanisms occurring in the cells. Individual cell models plug into the tissue scale models providing the ionic activity induced electrical current. Tissue‐scale organization is modeled depending on the organ, for example, stomach models possess three layers of smooth muscles (one longitudinal and two circular) separated by two ICC layers;^110^ whereas a small‐intestine model possesses only one smooth muscle cell layer and one ICC layer.^111^ Tissue‐scale electrophysiology modeling is based on a bi‐domain framework, which has previously been applied to cardiac electrophysiology^112^ and specifies the relationships between the potential difference across the cell membrane and the electric potential in the extra‐cellular space.

Thomas and coworkers proposed a whole modeling environment (SAPHIR) to model blood pressure regulation and fluid homeostasis.^113^ Chen *et al*. used a multiscale model to investigate the relationship between systolic blood pressure and the pathogenesis and progression of renal diseases.^114^

Computational oncology is another area where multiscale modeling is being used extensively. For a general review on the topic, see Deisboeck *et al*.^115^ May *et al*.^116^ coupled the biomechanics of tumor–host tissues interaction with a cellular model of cancer growth, an important determinant especially in tumors growing in regions confined by bone tissue, such as in the case of brain tumors; multiscale models are also used to investigate the role of angiogenesis in tumor growth,^72^ or to better understand the effect of radiotherapy.^117^

**Figure 10:**
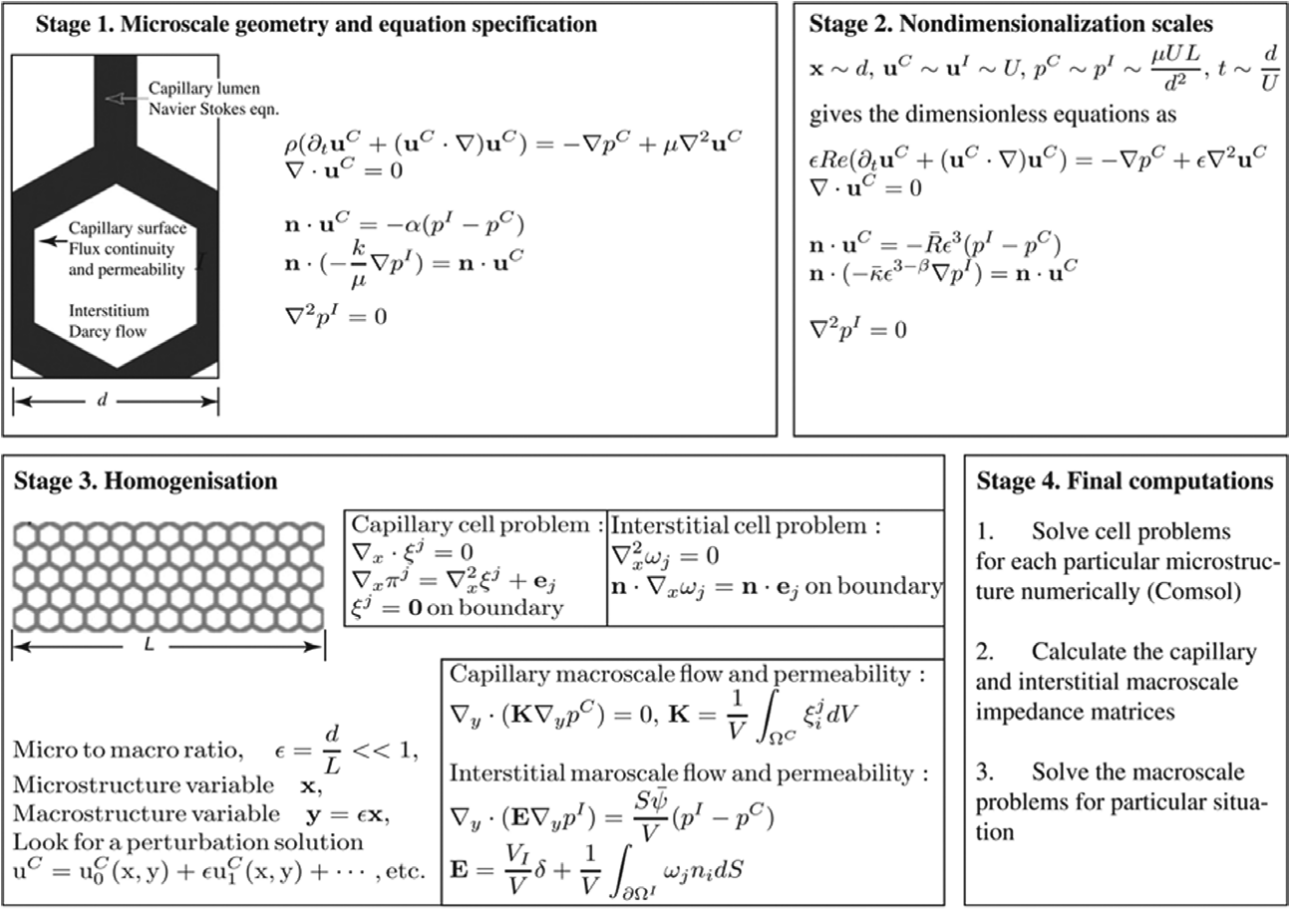
Multiscale modeling of lymphatic drainage. (Reprinted with permission from Ref 120. Copyright 2012 Elsevier)

Rim *et al*. used a three‐scale model to investigate the transdermal diffusion of drugs.^118^ Adra *et al*. developed an agent‐based model of keratinocyte colony formation in 2D culture.^119^ Roose and Swartz developed an extensive multiscale model of the fluid drainage from tissues through the lymphatic system (Figure 10).^120^ Biswas *et al*. developed a multiscale model of a skin mechanoreceptor, the Pacinian corpuscle.^121^ Valero *et al*. modeled the angiogenesis process during wound contraction.^122^

Another important application area is the modeling of organogenesis and growth processes. Newman *et al*. modeled the limb development process in vertebrates;^123^ Cox used a multiscale model to explore the mechanoregulation during dentition;^124^ Göktepe *et al*. proposed a multiscale model of the cardiac sarcomerogenesis.^125^

## CONCLUSION

Of all possible angles we could use to structure this review, we chose that of the modeling intent, organizing all reviewed papers around three distinct operational motivations: ‘Causal confirmation,’ ‘Predictive accuracy,’ and ‘Determination of effect.’ The first represents the most ‘humble’ intent, among the three. In these papers multiscale modeling is used to merely show that a complex set of observations is ‘compatible’ with a mechanistic theory embodied by the multiscale model; of course ‘compatible’ does not mean ‘true,’ and even less ‘accurate.’ The papers that fall in the predictive accuracy category are driven by the necessity to improve predictive accuracy over systems where the assumption of scale separation applies poorly. Last, the models motivated by the need to determine the effect of processes at radically different scale are the probably the most challenging, and intellectually interesting, as they look directly at the complexity of multiscale systems.

In the survey conducted here on the literature on multiscale modeling in biomechanics, we reviewed 72 studies motivated by causal confirmation, 12 studies aiming at predictive accuracy, and only one demonstrating determination of effect across scales (Table 3). Although the present review is not exhaustive, this relative multiplicity is representative of the body of research in the subdomain of multiscale modeling in biomechanics.

**Table 3:** Summary of the Reviewed Literature

Application Area	Causal Confirmation	Predictive Accuracy	Determination of Effect	All Categories
Musculoskeletal	20	8	0	28
Cardiovascular	30	4	1	35
Other	22	0	0	22
All areas	72	12	1	85

By focusing on the progress made beyond causal confirmation, we showed how in some research problems, a particular scale separation schema has gained wider acceptance through validation. For those topics where a valid scale separation picture is yet to emerge, this was found to be the case typically because model input variables/parameters at the different scales could not be determined in a specimen‐specific manner. This highlights focus areas for future experimental research. To clarify the road ahead, a total of 15 open problems (see Boxes 1 and 2) were posed in relation to musculoskeletal or cardiovascular applications which, if solved, would advance the state‐of‐the‐art of multiscale modeling in biomechanics. Finally, this review of current research reveals that, from basic biology to medicine, multiscale modeling in biomechanics is relevant to a variety of other research areas, and is expected to become more so in the future.

BOX 1 **OPEN PROBLEMS MOTIVATED BY PREDICTIVE ACCURACY**

1 **PA1**. Bone remodeling: A multiscale model to predict the evolution of bone mineral content as measured in a given bone volume, using bone remodeling activity parameters measured on the same specimen
2 **PA2**. Fracture healing: A multiscale model to predict tip cell movement and sprout formation as measured within a bone fracture site volume, using intracellular and tissue scale parameters measured on the same specimen
3 **PA3**. Skeletal muscle remodeling: A multiscale model to predict the shortening of the gastrocnemius muscle as measured on a subject, using sarcomere scale parameters measured on the same subject
4 **PA4**. Tendon homeostasis: A multiscale model to predict whole tendon remodeling as measured on a subject, using tissue and molecular scale measured on the same subject
5 **PA5**. Blood flow interaction with blood vessel walls: A multiscale model to predict the measured blood flow and blood vessel wall dynamics within a local region (e.g., surrounding an aneurysm) of the systemic circulation, using parameters measured on the same circulation system, vascular tissue and geometry and blood fluid
6 **PA6**. Blood flow interaction with blood coagulation: A multiscale model to predict the measured development of a blood clot, using the bio‐chemo‐mechanical parameters measured on the same platelet–blood–vessel wall system
7 **PA7**. Vasculogenesis and angiogenesis: A multiscale model to predict measured angiogenesis and vasculogenesis based on measured subcellular, cellular and molecular scale parameters
8 **PA8**. Cerebral autoregulation in CPB: A multiscale model to predict the measured cerebral autoregulation, using the measured elasticity of vessel walls on the same cardiac system
9 **PA9**. Cardiomyocyte mechanics: A multiscale model to predict the measured electrical activity in a cardiac myocyte under high‐frequency stimulation, using cell membrane electro‐mechanical property parameters measured in the same myocyte
10 **PA10**. Heart valve mechanics: A multiscale model to predict the change in cell aspect ratio during a cardiac cycle and increase in valvular tissue calcification due to aging, using cellular and tissue‐scale parameters measured in the same human heart valve

BOX 2 **OPEN PROBLEMS MOTIVATED BY DETERMINATION OF EFFECT**

1 **DE1**. Bone mechanics: A multiscale model that predicts better than a single‐scale model the bone tissue elastic anisotropy
2 **DE2**. Skeletal muscle electromechanics: A multiscale model that predicts better than a single‐scale model the force–length relationship in a muscle
3 **DE3**. Tendon tissue mechanics: A multiscale model that predicts better than a single‐scale model the stiffness tensor of a tendon tissue
4 **DE4**. Autonomic heart rate regulation: A multiscale model that predicts autonomic heart rate regulation under endotoxemic challenge better than a single‐scale model
5 **DE5**. Circulation system: A multiscale model that predicts the venous system in the head and in the neck better than a single‐scale model
